# Ileo‐rectal anastomosis in ulcerative colitis—Long‐term outcome, failure and risk of cancer at a tertiary centre

**DOI:** 10.1111/codi.16237

**Published:** 2022-07-11

**Authors:** John Berghog, Maria Hermanson, Hanna de la Croix, Jonas Bengtson, Mattias Block

**Affiliations:** ^1^ Department of Surgery Sahlgrenska University Hospital/Östra Gothenburg Sweden; ^2^ Institute of Clinical Sciences Sahlgrenska Academy Gothenburg University Gothenburg Sweden

**Keywords:** ileo‐rectal anastomosis, IBD‐surgery, ulcerative colitis

## Abstract

**Aim:**

Ileo‐rectal anastomosis (IRA) is an option to restore bowel continuity after colectomy in patients with ulcerative colitis (UC). Concerns that the remaining rectum may serve as a site for continuing proctitis with subsequent poor function and IRA failure and the fear of development of dysplasia and cancer have led to the abandonment of IRA in large parts of the world. This study investigated the outcome of IRA in a large patient cohort with UC and IRA with regard to failure of IRA and development of dysplasia and cancer.

**Methods:**

This was a retrospective data gathering of patients with UC and IRA enrolled at the Department of Colorectal Surgery, Surgical Clinic, Sahlgrenska University Hospital/Östra, Gothenburg, 1972–2019. End‐points were IRA failure, rectal dysplasia and cancer. IRA survival analysis and the cumulative probability of rectal cancer were calculated.

**Results:**

In total, 183 patients (121 men) were included in the study. The IRA failure rate was 34% and the estimated cumulative IRA failure rates were 25% and 35% at 5 and 10 years respectively. Four patients developed rectal cancer and the estimated cumulative probability of rectal cancer was 3% and 6% at 10 and 15 years respectively.

**Conclusion:**

Ileo‐rectal anastomosis remains a restorative option after colectomy for UC, even if the failure rate raises some concern. Further knowledge is needed for optimal patient selection to avoid early IRA failures. With increasing probability of rectal cancer over time a vigilant surveillance protocol is mandatory.

**What does this paper add to the literature?**

Ileo‐rectal anastomosis (IRA) after colectomy for ulcerative colitis is still performed at various centres; the literature on IRA in a modern setting is relatively sparse. We believe this paper adds further knowledge on IRA as a reconstructive method and that it can contribute to optimized patient selection for the procedure.

## INTRODUCTION

Despite advances in medical therapy, patients with ulcerative colitis (UC) still have an estimated 20‐year cumulative risk of colectomy of 15%–20% [1, 2, 3]. First described in the late 1970s, ileal pouch–anal anastomosis (IPAA) has become the method of choice for restorative surgery after colectomy for UC [4, 5, 6]. The major advantage of IPAA is that both the colon and rectum are resected, eliminating the need for anti‐inflammatory treatment and also eliminating the disease. The reported long‐term outcomes of IPAA vary, although quality of life seems to be the same as or slightly lower than that of the general population [7, 8, 9, 10].

However, the accumulated knowledge of IPAA has revealed certain drawbacks, mainly related to pelvic dissection, complications in the form of pelvic sepsis and pouchitis, sexual dysfunction, reduced fecundity in women, and pouch failure [11, 12, 13, 14].

Ileo‐rectal anastomosis (IRA) was advocated mainly by Aylett in the 1950s as a means to restore bowel continuity after colectomy [15, 16]. Compared to IPAA, IRA is generally regarded as a less complicated procedure without the need for pelvic dissection and therefore it entails a lower risk of associated complications.

Due to concerns regarding the risk for patients of developing cancer in the remaining rectum and persisting proctitis resulting in poor bowel function leading to IRA failure, IRA has been largely abandoned worldwide in favour of IPAA [17, 18]. However, it has been suggested that, when there is stringent patient selection, daily topical anti‐inflammatory treatment and regular endoscopic surveillance of the rectum, IRA represents either a permanent or interim alternative to IPAA [19, 20, 21].

A comparative analysis of patients with UC in England and Sweden found that IRA accounted for 59% of all restorative procedures in Sweden, compared to 7.7% in England [22]. At our institution, IRA has been an option in selected patients even if IPAA has reigned as the main restorative alternative. During the period of this study, 851 IPAA in patients with UC were constructed in comparison with 183 patients with IRA, accounting for 18% of the restorative procedures.

The aim of this study was to assess the long‐term outcomes for a large contemporary and consecutive cohort of individuals with UC who had undergone therapeutic colectomy and IRA, with the focus on postoperative complications, proctitis, failure rates and the development of rectal dysplasia and cancer.

## PATIENTS AND METHODS

Patients with UC who were operated with colectomy and IRA and attended an annual follow‐up at the Department of Surgery, Sahlgrenska University Hospital/Östra, during the period of 1972–2019 were included in the study. Clinical histories and data were retrieved retrospectively.

Criteria for patients eligible for IRA in our centre were and still are rectal‐sparing disease or mild inflammation at the most Mayo Grade 1, good/normal rectal compliance and no risk factors for developing dysplasia and/or cancer. Patients with a confirmed high grade of rectal inflammation, peri‐anal disease or dysplasia did not receive an IRA. Patients suitable for IRA were thoroughly informed about the importance of future surveillance and the mandatory use of topical agents. If the criteria for an IRA were met, the final decision of restorative method (IRA or IPAA) was made between surgeon and patient.

Accepting these terms, patients were scheduled for reconstructive surgery with an IRA.

Information regarding the preoperative endoscopic evaluations of the rectal mucosa was retrieved from the patients' medical charts and retrospectively graded according to the Mayo score [19] by the first author. In some patients' medical charts information about the macroscopic appearance of the rectal mucosa and presence of inflammation was missing and therefore listed as missing data.

Data regarding patients' characteristics, operating time, type of procedure (open vs. laparoscopic), length of hospital stay and perioperative blood loss were also retrieved.

Surgical complications were recorded and graded according to Clavien–Dindo [20].

The incidence rates of dysplasia and/or colorectal carcinoma were noted. Failure of the IRA was defined as deviation or resection of the rectal stump, with or without reconstructive surgery.

### Follow‐up

The Swedish follow‐up programme for UC with IRA includes annual monitoring of the remaining rectum. Patients with a duration of UC of more than 10 years, regardless of the date of their surgery, were followed up with annual clinical examinations, which included endoscopic assessment with multiple biopsies of the rectum to detect early signs of dysplasia and to assess the presence and extent of proctitis. All patients were prescribed and advised to use prophylactic 5‐aminosalicylic acid suppositories on a daily basis.

The patients were followed until failure of the IRA surgery, emigration from Sweden or death. Reasons for IRA failure were divided into three categories: persisting proctitis, poor function without proctitis and dysplasia/cancer.

For subgroup analysis, IRA failure was defined as early (within 2 years) or late (after 2 years) after IRA surgery.

To investigate if there were any differences in the numbers of IRA failures over time, the percentage of IRA failures in relation to the time period in which the IRA surgery was performed was noted. The total number of IPAAs performed in the corresponding time periods was also noted for reference.

The group of patients with IRA failure was compared to the non‐failure group with regard to the patients' characteristics, perioperative data and indication for colectomy. A further comparison with regard to the reasons for colectomy was made between the early and late failures.

### Statistical analysis

Patients' data were gathered retrospectively. Statistical analyses using Fisher's exact test and the Mann–Whitney *U* test were used for comparative analysis between the two groups of non‐failure and failure of ileo‐rectal anastomosis. A *P* value of ≤0.05 was considered significant. All data were checked for normality.

The survival rate of the patients who underwent IRA was calculated using a Kaplan–Meier curve. All calculations were performed using the IBM SPSS Statistics ver. 25 software.

## RESULTS

In total, 183 patients (121 men) with UC who were operated with a colectomy and reconstructed with an IRA were included in the study. The patients were followed at Sahlgrenska University Hospital, Gothenburg, Sweden. Sixteen (9%) of the patients underwent IRA surgery between 1972 and 1999 and the remaining patients (91%) between 2000 and 2019. The median follow‐up time was 6 (range 1–48) years.

**FIGURE 1:**
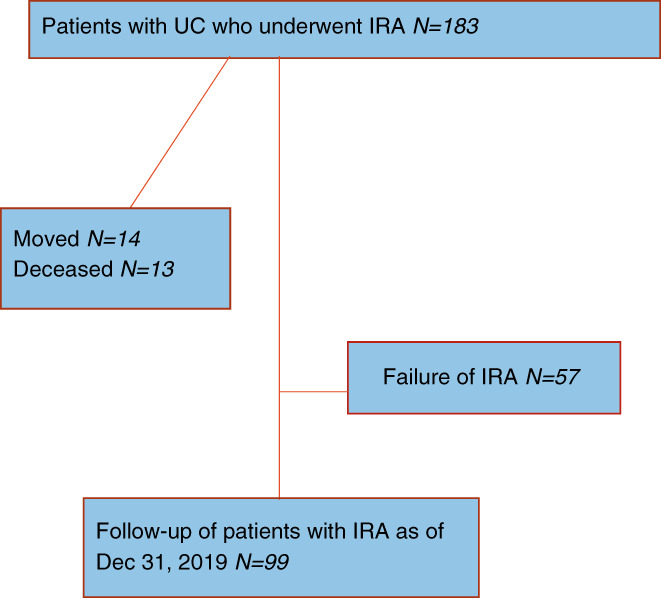
Overview of patients in the study cohort and patients remaining at follow‐up.

During the study period, 14 (8%) patients were lost to follow‐up because of emigration from Sweden, 13 (7%) patients died and 57 (34%) patients experienced a failure of their IRA (Figure 1). Eight patients out of 13 died from cancer: two from disseminated rectal cancer, one from cholangiocarcinoma and five from non‐UC‐related cancer. The remaining five patients died as a result of cardiovascular disease.

The median age at diagnosis was 25 (range 3–78) years, with a median age at colectomy of 30 (range 9–79) years and a median age at reconstructive surgery of 31 (range 11–80) years. The leading reason for colectomy was steroid dependence (56%) followed by acute colectomy due to acute severe colitis that did not respond to salvage therapy (35%). Other reasons for colectomy were dysplasia/cancer (7%): seven patients had cancer and the remainder had low grade dysplasia. All patients who had colectomy due to cancer had follow‐up regimens, and in select cases were given adjuvant chemotherapy in accordance with their tumour stage and treatment guidelines. At the end of cancer‐related follow‐up patients entered regular control with annual endoscopic examinations and mucosal biopsies after 10 years’ duration of UC.

Less common reasons for colectomy, for example colonic obstruction, accounted for 2%. A total of 45 (25%) patients had a primary IRA (at the same time as the colectomy). Most of the procedures were performed as open surgery, with 25 (14%) procedures being performed laparoscopically. The demographic characteristics of the patient population are described in Table 1.

**TABLE 1:** Characteristics of the ileo‐rectal anastomosis (IRA) cohort

IRA cohort	Total *N* = 183	Failure of IRA *N* = 57	Remaining IRA *N* = 126	*P* value
*N* (%)	121 (66)/62 (34)	37 (65)/20 (35)	84 (67)/42 (33)	0.867
Age, median (range), years; *N* = 177				
At diagnosis	25 (3–78)	25 (6–60)	25 (3–78)	
At colectomy	30 (9–79)	28 (9–61)	31 (11–79)	0.185
At reconstructive surgery	31 (11–80)	30 (11–63)	32 (14–80)	
Indication for colectomy *N* (%)				
Steroid dependence	93/166 (56)	29/53 (55)	64/113 (57)	0.482
Severe colitis	57/166 (35)	21/53 (40)	36/113 (32)	
Dysplasia/cancer	12/166 (7)	3/53 (5)	9/113 (8)	
Other^a^	4/166 (2)		4/113 (4)	
Missing	17/183	4/57 (7)	13/126 (10)	
Time to colectomy from diagnosis, median (range), years; *N* = 172	4 (0–35)	3 (0–35)	4 (0–30)	0.399
Time from colectomy to IRA, median (range), years; *N* = 178	0.8 (0–7)	0.9 (0–5)	0.7 (0–7)	0.317
Surgical procedure				
Open procedure *N* (%)	148/173 (86)	50/51 (98)	98/122 (80)	0.003
Laparoscopic procedure *N* (%)	25/173 (14)	1/51 (2)	24/122 (20)	
Missing		6/57 (10)	4/126 (3)	
Primary IRA *N* (%)	45 (25)	11 (19)	34 (26)	0.269
Operating time, median (range), min	156 (63–407)	158 (63–397)	155 (77–407)	0.991
Perioperative blood loss, median (range), ml	137 (0–4000)	150 (10–1600)	100 (10–4000)	0.681
Complications *N* (%)	39/158 (25)	10/47 (21)	29/111 (26)	
Clavien–Dindo				
I	13	4	9	0.959
II	10	1	9	
III	15	5	10	
IV	1	0	1	
Hospital stay (days)	7 (2–43)	6 (4–18)	7 (2–43)	0.067
PSC *N* (%)	17 (9)	10 (18)	7 (6)	0.010
Follow‐up time, median (range), years	6 (1–48)	3 (0–23)	9 (1–48)	<0.001

Abbreviation: PSC, primary sclerosing cholangitis.

^a^ Other indication for colectomy, including colon obstruction, perforation of the colon and two cases of polyposis of the colon (not familial adenomatous polyposis (FAP)).

### Dysplasia

Three patients had low‐grade dysplasia, one patient had high‐grade dysplasia and one patient was indefinite for dysplasia in mucosal biopsies at routine follow‐up and subsequently underwent proctectomy. However, the postoperative specimen revealed unambiguous dysplasia in two of these five patients. For one patient who had a duration of UC of 26 years and had IRA for 15 years, the postoperative specimen revealed minimal foci of low‐grade dysplasia. The other patient, who had a duration of UC and concomitant primary sclerosing cholangitis (PSC) of 13 years and had IRA for 3 years, presented with high‐grade dysplasia at routine follow‐up, and this was also confirmed in the postoperative specimen.

### Carcinoma

Four patients developed adenocarcinoma of the rectum. One 36‐year‐old patient who had a disease duration of UC of 24 years and had IRA for 7 years presented in an acute setting with peritonitis and sepsis and underwent emergency surgery. Further examination revealed Stage IV cancer and the patient later died from disseminated disease. A 34‐year‐old patient with a disease duration and IRA of 13 years also sought emergency care and was found to have advanced rectal cancer, resulting in small bowel obstruction. This patient underwent emergency surgery but later died due to Stage IV cancer and surgical complications. Unfortunately, due to poor patient compliance, both patients had missed out on many follow‐ups, including endoscopic evaluation, despite being sent several reminders by the clinic. Both patients had no other risk factor for developing rectal cancer than UC.

Two patients (aged 35 and 51 years, respectively) with disease duration of 8 and 19 years, respectively, and both with concomitant PSC developed rectal cancer which was detected during routine surveillance follow‐up. In the former case it was Stage III cancer and in the latter case it was Stage I cancer. Both patients underwent surgery with proctectomy, and the patient with Stage III cancer also received adjuvant chemotherapy. To date, these two patients show no signs of cancer recurrence. In the present study, the estimated cumulative probabilities of rectal cancer at 5, 10, 15 and 20 years were 1%, 3%, 6% and 6%, respectively. The median time from UC diagnosis to cancer‐related failure was 17 (range 14–24) years.

### Failure of the ileo‐rectal anastomosis

A total of 57 (34%) patients had failure of their IRA during the study period, with a median time from IRA to failure of 34 (range 1–278) months. The main reasons for failure were persisting proctitis in 32 (56%) patients, followed by poor function in 15 (26%) patients. A third group of nine (14%) patients had failure of their IRA due to suspected or confirmed dysplasia or carcinoma (as described above). One failure was due to a surgical complication that occurred only 2 days post‐surgery, and this case is not described further. A total of five patients (9%) had a Mayo score of 2 prior to IRA in the failure group compared to two patients (2%) in the non‐failure group (Table 2).

**TABLE 2:** Mayo scores prior to ileo‐rectal anastomosis (IRA) in the treatment non‐failure and failure groups

Mayo score	Non‐failure *N* (% of total)	Failure *N* (% of total)	*P* value
Normal	33 (26)	13 (23)	0.345
1	63 (50)	25 (44)	
2	2 (2)	5 (9)	0.027
Missing	28 (22)	14 (24)	
Total	126	57	

Fifty‐five per cent (24/57) of the failures due to persisting proctitis and poor function occurred within 2 years of the IRA procedure (Table 3). Failures due to the development of dysplasia and/or carcinoma generally occurred several years after the IRA procedure. Thirty‐eight (67%) patients with failure had undergone an IPAA procedure, while the remainder of the patients with failure had either a conventional (18/57, 31%) or a continent (1/57, 2%) ileostomy.

**TABLE 3:** Reasons for failure and time of failure (years) after ileo‐rectal anastomosis (IRA)

Reasons for failure	*N*	Years after IRA
Persisting proctitis	32	1, 1, 1, 1, 1, 1, 1, 1, 1, 1, 1, 1, 2, 2, 2, 2, 3, 3, 3, 3, 3, 4, 4, 4, 5, 5, 6, 6, 7, 9, 9, 11
Dysplasia/cancer	9	3, 4, **5, 7, 7, 13**, 16, 16, 23
Poor function	15	1, 1, 1, 1, 1, 1, 1, 1, 2, 3, 3, 10, 11, 13, 15

*Note*: The numbers in bold indicate cases of cancer.

**FIGURE 2:**
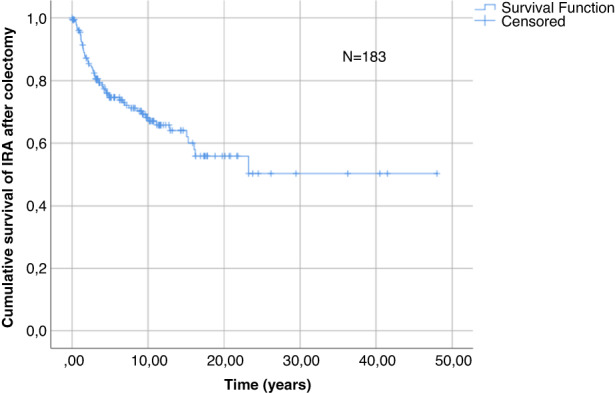
Cumulative survival curve for ileo‐rectal anastomosis (IRA) in the study cohort.

For the patients with UC who underwent an IRA, the estimated cumulative survival rates after 5, 10, 15 and 20 years were 75%, 67%, 62% and 56%, respectively (Figure 2).

### Non‐failure versus failure of ileo‐rectal anastomosis

Eighteen per cent (10/57) of the patients in the IRA failure group had concomitant PSC, compared with 6% (7/126) in the non‐failure group (*P* = 0.010; see Table 1). Nine per cent (5/57) of the patients in the IRA failure group had proctitis, Mayo Grade 2 according to preoperative endoscopic findings, compared to 2% (2/126) in the non‐failure group (*P* = 0.027; see Table 2). There were no other significant differences between patients who had a successful IRA and patients with IRA failure when considering the results of the preoperative endoscopic evaluation (Tables 1 and 2).

The major reason for performing colectomy in the early failure group was acute severe colitis (12/20) and the difference between groups in this regard was statistically significant (*P* = 0.023). Conversely, the leading reason for colectomy in the late failure group was steroid dependence (21/33; Table 4). A total of four patients (4/20) in the early failure group had a primary IRA compared to seven (7/33) in the late failure group; the difference was non‐significant (*P* = 0.971).

**TABLE 4:** Early versus late ileo‐rectal anastomosis (IRA) failure

Indication for colectomy	Early failure at 0–2 years post‐IRA; *N* = 20	Late failure > 2 years post‐IRA; *N* = 33	*P* value
Steroid dependence *N* (%)	8 (40)	21 (64)	0.154
Acute severe colitis *N* (%)	12 (60)	9 (27)	0.023
Dysplasia/cancer *N* (%)	0 (0)	3 (9)	0.282
Missing *N*	1	3	

The total number of IRA procedures performed during the assigned 5‐year intervals and the proportions of failed IRAs at each time interval are shown in Table 5. There were no significant differences in the failure rates between the time periods.

**TABLE 5:** Ileo‐rectal anastomosis (IRA) failures by time period and total numbers of IRA performed at each time period

IRA failures and numbers of IRAs performed per time period, *N* = 57				
	1972–2004	2005–2009	2010–2014	2015–2019
Failures at specific time period, *N* (% of total IRAs performed)	18/55 (33)	16/45 (35)	22/61 (36)	1/22 (4)
Number of IRA procedures per time period, *N* (%)	55/183 (30)	45/183 (25)	61/183 (33)	22/183 (12)
Number of IPAA procedures per time period, *N* (%)	572/851 (67)	50/851 (6)	80/851 (9)	149/851 (18)
Number of IPAA/IRA per time period, *N* (%)	572/627 (91) 55/627 (9)	50/95 (53) 45/95 (47)	80/141 (57) 61/141 (43)	149/171 (87) 22/171 (13)

Abbreviation: IPAA, ileal pouch–anal anastomosis.

## DISCUSSION

This is one of the largest single‐centre IRA cohorts studied since Aylett published his research on the feasibility of IRA in patients with UC [15, 16]. Previous studies have reported failure rates in the range of 13%–56% [21, 23, 24, 25]. In the present study, the failure rate of IRA was 34% (57/169), indicating a failure rate that agrees with those seen in previous studies. However, the failure rate of 34% is still in the higher range and improvement is feasible. Overall, 70% (40/57) of all failures occurred within 5 years of the IRA procedure. Of these, 20 patients had their failure within 2 years of the IRA procedure, possibly indicating poor patient selection in these cases, highlighting the importance of patient selection when IRA surgery is considered.

The estimated cumulative IRA failure rates at 5 and 10 years were 25% and 33%, respectively. These rates differ negatively from those of other studies showing cumulative 5‐year failure rates in the range of 10%–19% [21, 24]. The percentages of failures in relation to the number of procedures performed during specific time periods were essentially constant, indicating a weak impact of advances in medical therapy in recent years on the failure rates.

Our results underline the very important need for optimal patient selection criteria when deciding to perform IRA surgery. The associations between UC, concomitant PSC and high failure rates for IRA seen in the present study are supported by the findings from previous authors [26, 27]. Furthermore refractory proctitis not respondent to topical 5‐aminosalicylic acid treatment has previously been shown to predict worse outcome and higher risk for IRA failure [28]. Our study supports these results even if the number of patients in this group is very low.

In a subgroup analysis of the IRA failure group, a statistically significant percentage of the patients with early failure had severe colitis as an indication for colectomy. This finding, although confounding factors and the small sample size need to be taken into consideration, suggests that severe colitis as an indication for colectomy may serve as a risk factor for early IRA failure. Our findings differ from those of a retrospective multicentre study performed by Uzzan et al. who found colectomy on the basis of refractory UC to be a risk for IRA failure [28]. The potential correlation between IRA failure and indication for colectomy needs to be studied further.

Although found to be low in this study, the risk of developing cancer of the rectum with a potentially disastrous outcome for the patients remains a major drawback associated with IRA. In our patient cohort, four patients had adenocarcinoma of the rectum that resulted in an estimated cumulative probability of rectal cancer at 20 years of 6%, which is in line with or even lower than the results of previous studies [21, 24, 29].

In previous studies, concomitant PSC has been reported to be a risk factor for developing colorectal cancer in patients with an IRA [29, 30]. In a multicentre, retrospective study of 343 patients with UC who underwent IRA surgery, Uzzan et al. reported the duration of UC and concomitant PSC as one independent risk factor for developing rectal cancer, and in a recent study Lundberg et al. reported an increased risk of colorectal cancer in patients with PSC with a hazard ratio of 7.5 [29, 31]. So, taking into account the increased risk of rectal cancer in patients with PSC UC, the increased risk for poor function and the risk for failure of the IRA, we strongly suggest that concomitant PSC should be considered as a contraindication for IRA.

Two patients out of four who developed rectal cancer in our study had concomitant PSC, so our results agree with the findings of these previous studies [29, 30, 31] . The median age at the time of the IRA procedure was 31 years and, given that the overall life expectancy of the UC population mirrors that of the general population [32], this inevitably leads to many patient‐years at risk of rectal cancer. However, the risk of developing rectal cancer for patients who undergo IRA also needs to be seen in the light of the overall probability of developing colorectal cancer in the UC population as a whole, whereby the reported 20‐year cumulative probability varies between 1.5% and 8.0% [33, 34].The discrepancy observed between real dysplasia in postoperative specimens and in the mucosal biopsies prior to proctectomy in the five patients who had IRA failure on the basis of suspected dysplasia is a further reminder of the difficulties associated with histopathological differentiation of inflammatory changes from dysplasia [35, 36]. That two patients died from rectal cancer in our study highlights the utmost importance of annual clinical examinations, as well as patient compliance to attend these appointments, in the light of a disease duration of UC of ≥10 years. The increased risks of dysplasia and cancer over time lend support to the viewpoint put forward by other authors that IRA may work as an interim solution for a select group of predominantly young patients, given the risks of sexual dysfunction and infertility linked to IPAA [13, 21, 24].

### Strengths and weaknesses of the study

A major strength of this study is its sample size. This is one of the largest single‐centre IRA cohorts studied since the 1950s [16].

There are some limitations to the study. The major limitation is the retrospective study design with its inherent shortcomings. Another weakness, in conjunction with the retrospective study design, is that the study lacks the medical treatment history of patients; changes in the handling of medical charts from paper to electronic or missing data have in many cases made the full medical treatment of patients hard to obtain. In this study, however, we saw no decline in IRA failure rates after the introduction of anti‐tumour necrosis factor α therapy.

The long study period of this study is in one way a weakness but could also serve as a strength. The long study period entails that some patients received their IRA before the age of biological agents, and perhaps patients at that time had milder disease to be considered for an IRA. The majority of patients in this study were operated after 2005, well into the age of biological agents, making it possible to also contrast IRA failure in the pre‐biological and biological era, and the long time frame of the study in this instance serves as a strength in our opinion.

This study did not take into account IRA function, which is an important parameter when studying patients who have undergone an IRA. Nevertheless, previous studies have shown that long‐term bowel function following IRA is at a satisfactory level and similar to that achieved with IPAA [25, 37, 38].

## CONCLUSION AND FURTHER STUDIES

Ileo‐rectal anastomosis remains a restorative option after colectomy for the treatment of UC; the reported failure rate of 34% and the relatively high number of patients with IRA failure within 2 years of surgery is problematic and serves as a powerful reminder of the importance of proper patient selection for the IRA procedure. Concomitant PSC is a risk factor for IRA failure and also brings a higher risk of developing dysplasia and/or carcinoma. PSC should therefore be regarded as a contraindication for IRA. Follow‐up endoscopy is mandatory after IRA and the understanding, willingness and ability of patients to take part in such annual follow‐ups needs to be considered carefully when deciding on the optimal restorative procedure.

## AUTHOR CONTRIBUTIONS

John Berghog contributed to the conception, design, acquisition of data, analysis and interpretation of data and drafting of the study. Maria Hermanson contributed to the analysis and interpretation of data and drafting of the study. Hanna de la Croix contributed to the analysis and interpretation of data and drafting of the study. Jonas Bengtson contributed to the analysis and interpretation of data and drafting of the study. Mattias Block contributed to the conception, design, analysis and interpretation of data and drafting of the study.

## FUNDING INFORMATION

The study was supported by grants from the Anna‐Lisa and Bror Björnssons Foundation and from Sahlgrenska University Hospital (Agreement regarding Doctor Education and Research; ALF‐GBG‐773521).

## CONFLICT OF INTEREST

The authors have no conflicts of interest to declare.

## ETHICS STATEMENT

The study was approved by the Swedish Ethical Review Authority (registration number 2021‐00380).

## Data Availability

The data underlying this article cannot be shared publicly due to the privacy of individuals that participated in the study. The data will be shared on reasonable request to the corresponding author.
